# The Interconnecting Process and Sensing Performance of Stretchable Hybrid Electronic Yarn for Body Temperature Monitoring

**DOI:** 10.3390/polym16020243

**Published:** 2024-01-15

**Authors:** Fenye Meng, Shaoqing Dai, Yong Zhang, Jiyong Hu

**Affiliations:** 1 School of Fashion & Art Design, Jiaxing Vocational & Technical College, Jiaxing 314036, China; mengfy@jxvtc.edu.cn (F.M.);; 2 Shanghai Frontiers Science Center of Advanced Textiles, Donghua University, Shanghai 201620, China

**Keywords:** hybrid electronic yarn, stretchable, temperature sensing, interconnection, encapsulating

## Abstract

Flexible and stretchable electronic yarn containing electronic components (i.e., hybrid electronic yarn) are essential for manufacturing smart textile garments or fabrics. Due to their low stretchability and easy interconnection fracture, previously reported hybrid electronic sensing yarns have poor mechanical durability and washability. In order to address this issue, a stretchable hybrid electronic yarn for body temperature monitoring was designed and prepared using a spandex filament as the core yarn and a thin enameled copper wire connected with a thermal resistor as the wrapping fiber. The temperature sensing performance of different hybrid electronic yarn samples was evaluated using the following three types of interconnection methods: conductive adhesive bonding, melt soldering, and hot pressure bonding. The optimal interconnection method with good sensing performance was determined. Furthermore, in order to improve the mechanical durability of the hybrid electronic yarn made using the optimal interconnection method, the interconnection area was encapsulated with polymers, and the effect of polymer materials and structures on the temperature-sensing properties was evaluated. The results show that traditional wrapping combined with hot pressing interconnection followed by tube encapsulating technology is beneficial for achieving high stretchability and good temperature-sensing performance of hybrid electronic yarn.

## 1. Introduction

Body temperature is a vital indicator of human health, and its abnormal changes usually indicate poor health. The real-time and continuous monitoring of body temperature is particularly important for disease prevention and treatment [1,2]. Traditional temperature sensors were too bulky and difficult to integrate directly into clothing, resulting in an unsatisfactory wearing experience and visual aesthetics [3]. Therefore, achieving the continuous and unobtrusive monitoring of body temperature remains a technological challenge [4,5].

Temperature-sensing yarn is a new type of flexible functional yarn that enables the unobtrusive monitoring of body temperature. Typically, temperature sensing-yarn can be classified into the following three types based on the integration mode of thermosensitive material into fiber/yarn [6]: (1) thermosensitive fibers such as platinum wire and optical fiber are large in size and low in stretchability, making it difficult to measure multiple local points when worn; (2) fibers or yarns deposited by thermosensitive materials, such as coated thermosensitive ink or sputtered metal film, are prone to cracking or peeling off under tensile and bending deformations; (3) hybrid fiber or yarn embedded with mini-temperature sensors, i.e., hybrid electronic yarn, are prone to wear and tear in their interconnected segments.

Recently, the hybrid electronic yarn that integrates micro-thermistors [7,8] or micro-LED [9] and regular textile yarn has attracted extensive attention. This type of temperature-sensing yarn is superior in its excellent compatibility with bodysuits and its capacity to continuously record multi-point body temperature due to the micro-thermistor [10,11]. Some studies have discussed the manufacturing technology and temperature sensing performance of hybrid electronic yarn using straight copper wire, and it was observed that there was a fracture at the soldering and encapsulated area after washing [7]. This fracture can be attributed to the poor stretchability of the hybrid electronic yarn. Alternatively, a combination of multi-strand copper wires twisted together with three cotton yarns was used to connect the thermistor and make it endure a 50% stretching strain. However, it was reported that under no tension, the resistance decreased by nearly 40% compared to when the yarn was under tension, resulting in a measurement error of 5.9 °C [12]. Therefore, new interconnection structures and encapsulating methods need to be developed in order to improve the durability and maintain sensing accuracy for this kind of hybrid electronic yarn. Previous work has also shown that the helix structure is a common strategy for achieving the good stretchability of metal wire [13,14,15] while bonding technology with a conductive adhesive is a mature and applicable interconnection technology for producing printed flexible hybrid electronics [16]. In this way, the strategy combining the helix wire structure with bonding technology could potentially enable high mechanical durability and large-scale manufacturing for hybrid electronic yarn and then improve human motion flexibility, the working performance stability, and the service lifetime of smart garments or fabrics.

This work prepares hybrid electronic temperature sensing (HETS) yarn with a helix wire structure using different interconnection and encapsulation technologies. The effects of its interconnection and encapsulation on the temperature sensing properties, as well as the bending failure behavior, are evaluated to determine the optimal technology. The development of HETS yarn could provide an alternative tool for monitoring distributed body temperature in wearable smart clothing.

## 2. Material and Experiment

### 2.1. Structure Design of HETS Yarn

**Figure 1:**
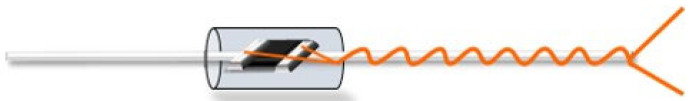
Structure illustration of stretchable HETS yarn.

The structure of the stretchable HETS yarn is shown in Figure 1. Here, two thin enameled copper wires as signal transmission lines were helically wrapped around the elastic yarn/fiber as carrier yarn, and two contactors of the micro-thermistor were connected to one end of each of the two wires, respectively. The interconnection area was encapsulated to improve mechanical durability and insulation as well as water resistance.

### 2.2. Materials

The signal transmission lines of the micro-thermistor were made using a pair of enameled copper wires with a diameter of 0.07 mm (Hongda Electronics Co., Ltd., Dongguan, China), which possess good electrical conductivity, thermal conductivity, and water resistance. The carrier yarn used was a high elastic spandex filament with a finesse of 1120 D. For the temperature sensing unit of the hybrid electronic yarn, a commercial 0402 NTC (negative temperature coefficients) micro-thermistor (10 KΩ, 1.0 mm × 0.5 mm × 0.5 mm, *β* = 4050) (Ruiside technology, Co., Ltd., Shenzhen, China) was selected. The following two types of common interconnection bonding materials were utilized: lead-free solder paste (with a melting point of 138℃, Jiede New Material Co., Ltd., Shenzhen, China) and a two-component copper isotropic conductive adhesive (DB2011, Xinwe Material Co., Ltd., Shenzhen, China). To insulate the interconnection area formed by the micro-thermistor and hybrid electronic yarn, the following four types of previously reported encapsulation materials were applied: polyurethane acrylate (PUA) (Guangju Technology Co., Ltd., Chengdu, China), PDMS, Ecoflex00-50 (Sinopharm Chemical Reagent Co. Ltd., Shanghai, China), and a transparent heat-shrinkable polymer tube with an inner diameter of 0.8 mm (Kaiheng Enterprise Group Co., Ltd., Guangzhou, China) with an outer diameter of 1.4 mm and length of 2.0 mm [17,18]. The oven and hot presser (F-320) were employed to cure both the interconnection and encapsulating materials.

### 2.3. Stretchable HETS Yarn Preparation

The preparation of stretchable HETS yarn mainly involves three steps. (1) The stretchable composite yarn is prepared by wrapping two parallel copper wires around the elastic spandex filament. A Hollow Spindle Spinning Frame (provided by Jinggong Machine Co., Ltd., Sizhou, China) was used to wrap the copper wire around the spandex filaments with 1800 twists/m. (2) Hybrid integration was achieved by connecting two copper wires to each of the two contactors of the NTC thermistor with the following interconnection process, respectively. (3) The interconnection area is encapsulated by the above-mentioned four types of insulating materials.

#### 2.3.1. Interconnection Process

**Figure 2:**
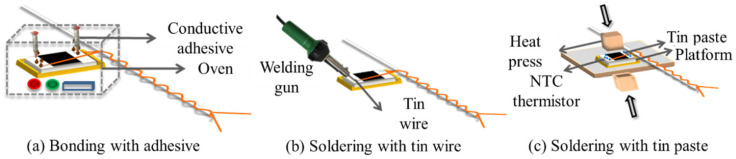
Interconnection methods for stretchable hybrid electronic yarn and the NTC thermistor.

The interconnection between the NTC thermistor and the enameled copper wires of hybrid electronic yarn is not only relevant to the accuracy of temperature sensing and signal transmission but also determines the service life of HETS yarn. In this experiment, as shown in Figure 2, three types of easy-to-operate and cost-effective interconnection technologies were used to connect the thermistor with a pair of copper wires of stretchable hybrid electronic yarn.

##### Bonding with Conductive Copper Adhesive

Two-component copper ICA was chosen as the conductive adhesive for interconnecting the thermistor and electronic wires in surface mount technology [13,17]. The NTC thermistor was securely positioned in a customized groove mold, with its two contactors connected to the enameled copper wires using a drop of DB2011, a two-component copper ICA. Subsequently, it underwent heating at 80 °C for 10 min in an oven to complete the bonding process, as illustrated in Figure 2a.

##### Soldering with a Lead-Free Tin Wire

The NTC thermistor and the enameled copper wires were connected by soldering the lead-free tin wire with a 30 W Internal Heat Welding Gun at high temperature with reference to previous work [19], as depicted in Figure 2b.

##### Soldering with a Lead-Free Tin Paste

The NTC thermistor was inserted into the customized groove mold on a 4 cm × 4 cm platform. A low-temperature lead-free soldering paste (with a melting point of 138 °C) was applied to the interface between the enameled copper wire and the NTC thermistor. Subsequently, the F-320 heat presser with a rectangle size of 1 cm × 1 cm under 0.7 N force was placed on top and held at 165 °C for 10 s [16], as depicted in Figure 2c.

#### 2.3.2. Encapsulation Process

**Figure 3:**
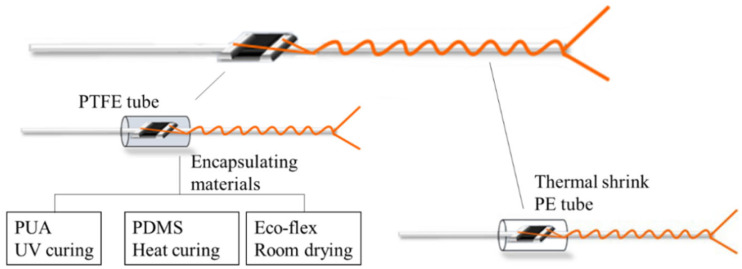
Encapsulation illustration of interconnection area.

The functional components of stretchable HETS yarn consist of micro NTC thermistors and enameled copper wires. When exposed to environmental erosion and mechanical actions during manufacturing processes or service life, cracks may occur, leading to sensing failure. Therefore, it is necessary to protect the functional components in order to enhance their durability against external forces such as friction, moisture, sweat, and water. Based on previously reported common failure modes [5,7], encapsulation can only be applied to the interconnection area of stretchable HETS yarns that exhibit good sensing performance, as shown in Figure 3. Meanwhile, the encapsulation process can only be applied to HETS yarn with the optimal interconnection.

The encapsulated interconnection area of the NTC thermistor and enameled copper wire should be minimized to maintain flexibility and invisibility and improve the wearable comfort of the integrated product. Therefore, a transparent and hollow polytetrafluoroethylene (PTFE) tube with an outer diameter of 1.4 mm and length of 2 mm (Jinda fluorine plastic Co., Ltd., Wenzhou, China) was selected as the encapsulating mold in this experiment. Additionally, the PTFE tube was easily demolded after curing. The UV-curing polyurethane acrylate (PUA) [17], thermal-curing polydimethicone (PDMS with prepolymers: hardeners, 10:1) [20], and silicone Eco-flex (silicone resin: platinum, 1:1) were used as the encapsulation material, respectively. Firstly, the interconnection area of the NTC thermistor and wire was covered using the PTFE tube; then, different encapsulation materials were injected into the PTFE tube and cured accordingly: PUA was cured for 40 s under UV light (xenon lamp, 1700 w); the PDMS solution was heated at 80 °C for 1 h in an oven; and Eco-flex was dried horizontally at room temperature (25 °C, 65%RH) for 30 min.

To provide a comparison, a heat-shrinkable polyester (HSP) tube with a shrinking ratio of 3:1 was utilized to encase the interconnection area using a heating gun at 120 °C for 10 s. The dimensions of the HSP tube were identical to those of the transparent PTFE tube.

#### 2.3.3. Test and Evaluation

The impact of interconnect technology, including two-component conductive adhesive bonding, melt soldering, and heat pressure soldering, on the properties of stretchable HETS yarn, were assessed in terms of surface morphology, electrical resistance variation, sensitivity, bending fatigue, and the encapsulation effect to identify the optimal interconnect method. To note, this work does not evaluate the stretchability in terms of the intrinsic large strain of the helically wrapping structure of the HETS yarn, and also washability due to multiple factor effects.

(1) Surface morphology

The surface morphology of the interconnect area between the NTC thermistor and copper enameled wire was observed using an HDMI200C-B Electron Microscope (Niuman, Shenzhen, China). It is desirable for the interconnection area to be as small as possible in order to minimize the presence of hard regions and enhance the overall flexibility of stretchable HETS yarn.

(2) Electrical resistance variation

A 2 m long HETS yarn was placed flat in a serpentine manner on the 10 cm × 10 cm heating plate with the ends connected to the two-wire resistance port of Agilent 34970A (Agilent, CA, USA). The electrical resistance of the NTC thermoresistor was tested by a pair of probes contacting two contactors of the thermoresistor, respectively. Then, the resistance of the copper wire was the difference between the HETS yarn and thermoresistor. Its electrical resistance was measured in a standard lab to ensure the test’s stability. The test temperature was adjusted by the heating plate within 28–43 °C (human body temperature range). The resistance of the HETS yarn was measured at 1.0 Hz when the temperature increased by 1 °C at each time and held for 5 min; then, data were continuously collected for 2 min and averaged among five repeated samples.

(3) Sensitivity

The temperature coefficient of resistance (TCR) [21], which represents the sensing sensitivity and, namely, the ratio of the relative electrical resistance change to the temperature change, was determined using Equation (1).

(1) $$TCR(\alpha )=\frac{R_{T}-R_{0}}{R_{T}(∆T)}$$

where *R*_T_ is the resistance at *T* °C; *R*_0_ is the initial resistance of the sample at *T*_0_ °C; Δ*T* is the temperature change step; and *T = T*_0_ *+* Δ*T*.

The hysteresis phenomenon is commonly employed to indicate the sensing response error during temperature variations. The hysteresis effects of HETS yarn were evaluated by calculating the hysteresis error ${(\delta }_{h})$ using Equation (2) [22]. This error was defined as the maximum electrical resistance difference between the heating and cooling stages corresponding to the same temperature within one cycle.

(2) $$\delta_{h}=\frac{{\left(∆R\right)}_{max}}{R_{H}-R_{C}}\times 100\%$$

where (Δ*R*)*_max_* is the maximum difference in electrical resistance between the heating and cooling process, corresponding to the same temperature within one heating–cooling cycle (25–45 °C), *R_H_*_,_ *R_C_* is the electrical resistance at *T*_h_ °C during heating and cooling.

**Figure 4:**
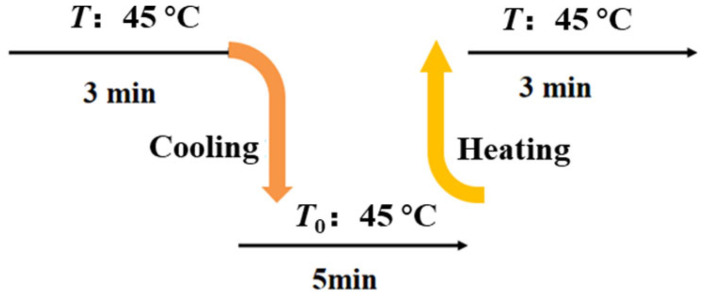
Testing scheme illustration for the response time of TSHE yarn.

The response time was usually utilized to indicate the response speed of HETS yarn to temperature variations, representing the duration required for it to reach a stable final value [7]. Generally, it refers to the time taken by the temperature sensor for a 63.2% change in temperature [23]. In this experiment, the response time was measured using the testing procedure depicted in Figure 4. The resistance changes were recorded following heating and natural cooling processes under a frequency of 1.0 kHz, with six repeated tests conducted on each sample. Five samples were prepared using different interconnection methods, and their average values were calculated.

(4) Bending fatigue

Stretchable HETS yarn is likely to undergo bending action in practical applications, which may result in the fracture of the interconnection area after multiple bending cycles and lead to the dysfunction of HETS yarn. HETS yarn has the best sensitivity among the three kinds of interconnection methods; therefore, an evaluation was conducted on the bending fatigue of HETS yarn by wrapping it around a rigid cylinder with a diameter of 18 mm and subjecting it to 100 bending/flattening cycles. HETS yarn with a length of 10 cm and the thermistor 5 cm away from the yarn end was prepared. During testing with a customized program rotator, the thermistor of HETS yarn was at the middle line of the rigid cylinder; one end of HETS yarn was fixed, and the other was firstly rotated around the cylinder by 90° and then returned to the horizontal level. The electrical resistance value at room temperature (25 °C) was recorded after each cycle using the Agilent 34970A. The changes in electrical resistance before and after bending were calculated.

(5) Encapsulation effect

For each of the samples prepared using different encapsulating methods, the sensing sensitivity, the maximum hysteresis error, and the response time of four types of HETS yarn after the encapsulation process were evaluated using the aforementioned testing methods.

## 3. Results and Discussion

### 3.1. Temperature–Resistance Response of Hybrid Electronic Yarn

**Figure 5:**
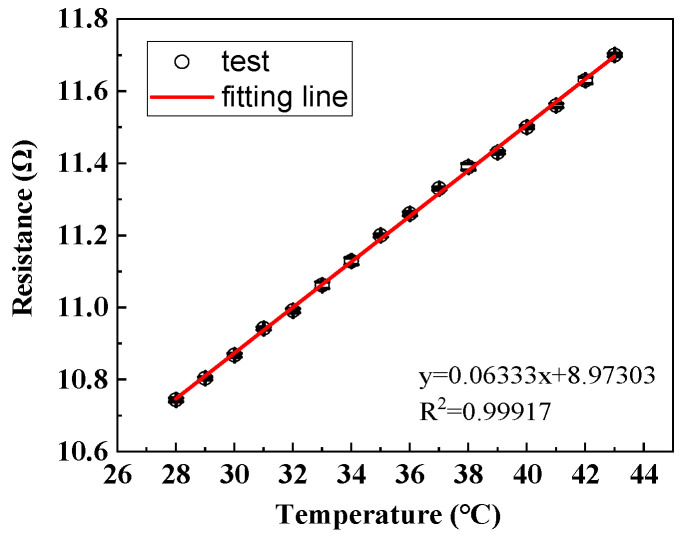
The resistance-temperature change in hybrid electronic yarn.

The measured electrical resistance of stretchable hybrid electronic yarn before encapsulation within the 28–43 °C range was depicted in Figure 5. It can be observed that the resistance exhibited a linear increase with rising temperatures, albeit at a significantly low magnitude at merely one-thousandth of the NTC thermistors. At its peak, the maximum resistance reached approximately 12 Ω at 43 °C. Furthermore, for every increment of 1 °C, there was a corresponding change in the copper wire resistance by only 0.077 Ω, which accounted for just 0.024% of the overall variation observed in the measured resistance of hybrid electronic yarn. Consequently, it can be concluded that the electrical resistance of enameled copper wire displayed relatively low sensitivity to environmental temperatures, and any potential impact on sensing performance could be disregarded.

### 3.2. Sensing Performance of HETS Yarn

(1) Sensitivity

**Figure 6:**
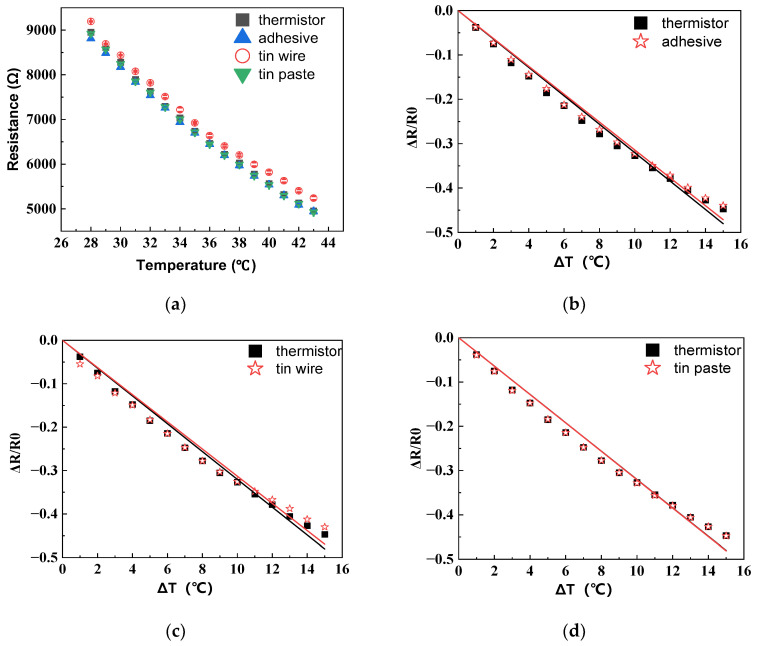
Temperature–resistance responses of HETS yarn with three different interconnection methods. (**a**) Resistance–temperature change; (**b**) relative change for conductive adhesive bonding; (**c**) relative change for soldering with tin wire; (**d**) relative change for soldering with tin paste.

The temperature–resistance change in the HETS yarn, prepared using different interconnection technologies, is illustrated in Figure 6a. The resistance exhibits a significant linear decrease with an increasing temperature (R^2^ ≥ 0.99), indicating that three methods for connecting the stretchable hybrid electronic yarn and NTC thermistor were viable. Notably, soldering with tin paste demonstrates the smallest deviation in fitting the temperature–resistance change in HETS yarn compared to the original NTC thermistor, as depicted in Figure 6b–d.

**Figure 7:**
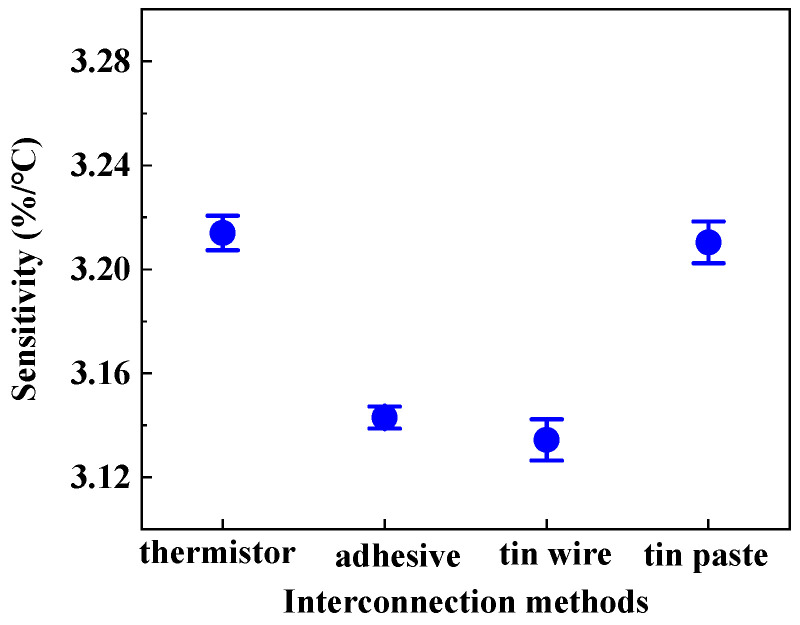
Sensitivity of HETS yarn prepared using three different interconnection methods.

The sensitivity of HETS yarn was determined by the slope of the electrical temperature–resistance change curve. Figure 7 illustrates the sensitivity of HETS yarn prepared using three different connection methods, along with a comparison to the response of an NTC thermistor. The NTC thermistor exhibited the highest sensitivity (3.214%/°C), which decreased by 2.48% (3.134%/°C) after being connected with enameled copper wire. All three samples of HETS yarn demonstrated sensitivities exceeding 3.13%/°C, significantly surpassing that reported for super elastic temperature-sensing fiber (≈0.93%/°C) [17]. This indicates that all three interconnecting methods employed were viable in fabricating HETS yarn.

The sensitivity of HETS yarn obtained through soldering with tin paste (3.210%/°C) closely approximated that of the NTC thermistor due to the effective formation of a secure and stable contact between the wire and thermistor, resulting in reduced additional resistance. Additionally, the electrical resistance changes in the 2 m long conductive composite yarn were only 0.592%/°C, which is merely 0.024% compared to that of HETS yarn, rendering the temperature effect negligible when evaluating the sensing performance of HETS yarn.

(2) Hysteresis

**Figure 8:**
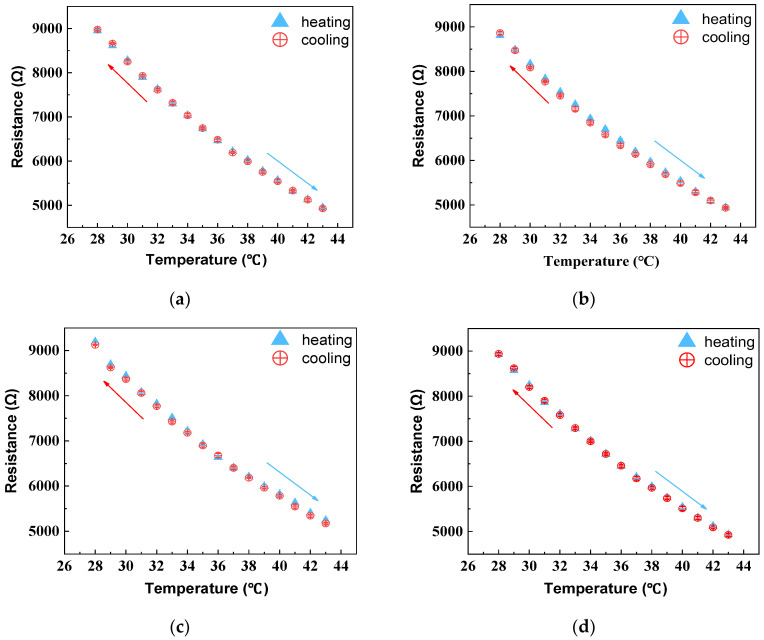
Temperature–resistance relationship of thermistor and sensing yarns with three interconnection methods under heating–cooling cycles. (**a**) thermistor; (**b**) conductive adhesive bonding; (**c**) melt soldering with tin wire; (**d**) heat pressing soldering with tin paste. The arrows indicate the cooling or heating direction.

The largest resistance change in the sample during the heating and natural cooling test cycles was recorded and depicted in Figure 8. The temperature–resistance curves of HETS yarn during the ascending and descending cycle did not completely coincide, with HETS yarn bonded using a conductive adhesive exhibiting a maximum resistance error of 153 Ω. According to Equation (2), the maximum hysteresis error of the NTC thermistor was calculated at 1.17%, while the hysteresis error for HETS yarn prepared through heat-pressing soldering with tin paste was only 1.33%, a mere increase of 0.16% compared to that of the NTC thermistor. However, when comparing it with two-component conductive adhesive bonding and melt soldering with tin wire methods, both exhibited hysteresis errors that were approximately 2.6 times larger than that observed in thermistors due to their utilization of thicker soldering materials. In comparison to previously reported results [22], all three interconnection methods used in this study resulted in sensing yarns with lower hysteresis effects, which were smaller than those reported (3.65% and 3.26%, respectively). Therefore, it can be concluded that stretchable HETS yarns developed in this work exhibit reduced hysteresis effects.

(3) Response time

**Figure 9:**
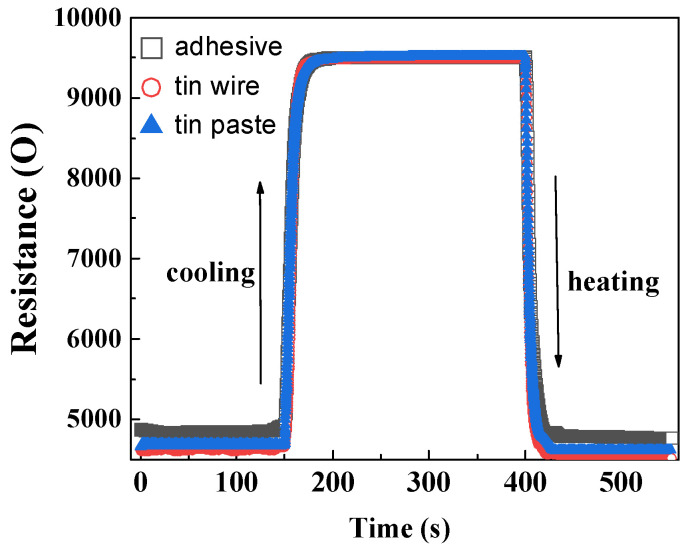
Dynamic resistance responses of HETS yarn with different interconnection methods.

The dynamic response of HETS yarns prepared using different interconnection methods at room temperature and during controlled heating and natural cooling is shown in Figure 9. The three types of HETS yarn exhibited varying electrical resistance at the same heating temperature, with a maximum resistance difference of 250 Ω observed at approximately 0.8 °C. The resistance responses of the samples during step heating start and stop were rapid, with the heating response being faster than the cooling response, as indicated by the slope of the curve in Figure 8. The resistance stabilized after 25 s during natural cooling, whereas it only took 15 s during step heating.

The response time, which represents the time for 63.2% of the resistance change, was calculated and is recorded in Table 1. The calculated response time of the sensing yarns using three different interconnection methods exhibited consistency with the slope of the curve shown in Figure 8. Additionally, it was observed that during cooling, the response time was approximately 0.65–1.35 s slower compared to heating. This phenomenon can be explained by active heat absorption during heating and passive heat dissipation during cooling. Notably, when comparing the three interconnection methods, it was found that soldering tin wire provided a metal heat transmission path, resulting in a shorter response time during heating. Considering how human body temperature monitoring usually takes several minutes and even hours, a small response time difference of less than 1 s is negligible.

(4) Bending fatigue

**Figure 10:**
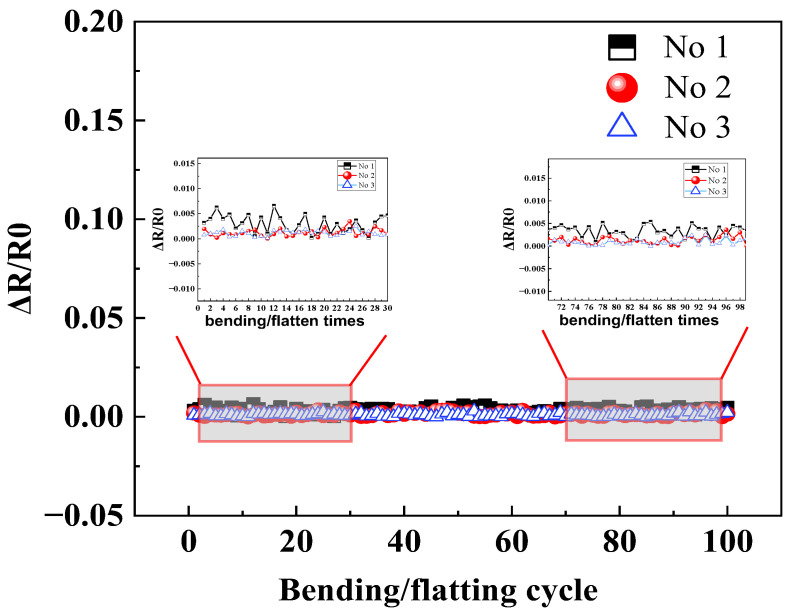
Relative resistance change in HETS yarn with heat pressing soldering after 100 bending/flattening cycles.

By conducting a total comparison of the sensitivity, max hysteresis error, and response time, tin paste soldering among three interconnection methods is the optimal technology, and then the bending fatigue test was applied for HETS yarn using the tin paste soldering interconnection method. The hybrid yarn produced by tin paste soldering exhibited superior sensing properties, as evidenced by its resistance change after the 100 bending/flattening cycles shown in Figure 10. The relative resistance change remained minimal even after undergoing 100 bending/flatting cycles, which is consistent with the findings reported [10]. This observation suggests that HETS yarn with tin paste soldering demonstrates enhanced mechanical endurance performance during wear and washing; herewith, the following work discusses the encapsulation effect of HETS yarn with tin paste soldering.

## 4. Encapsulation Effect

### 4.1. Surface Morphology

**Figure 11:**
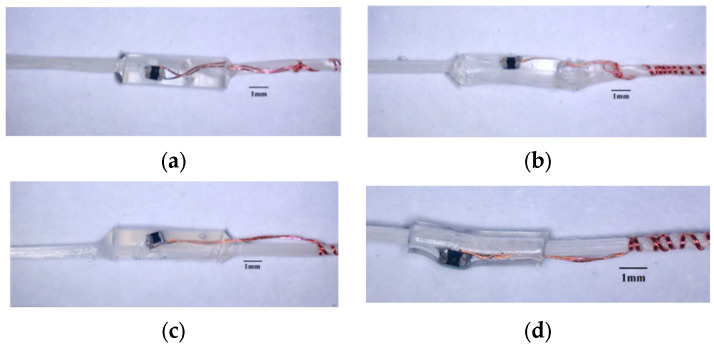
The morphology of interconnection area encapsulated with different materials. (**a**) PUA; (**b**) PDMS; (**c**) Ecoflex; (**d**) heating shrinkable tube.

For HETS yarn with tin paste soldering, the surface morphologies of different encapsulating materials are illustrated in Figure 11. It is evident that the encapsulating body ends with PUA, and Ecoflex and Ecoflex material exhibits a gradual transition due to the rheological behavior of the injected encapsulating materials. This transition is advantageous in alleviating the stress concentration effect at the interconnection interface during bending. In contrast, the interconnection area enclosed by a heating shrinkable tube features a sharp edge, which easily leads to stress concentration and wire fracture during cyclic bending.

### 4.2. Sensing Property of HETS Yarn with Encapsulation

**Figure 12:**
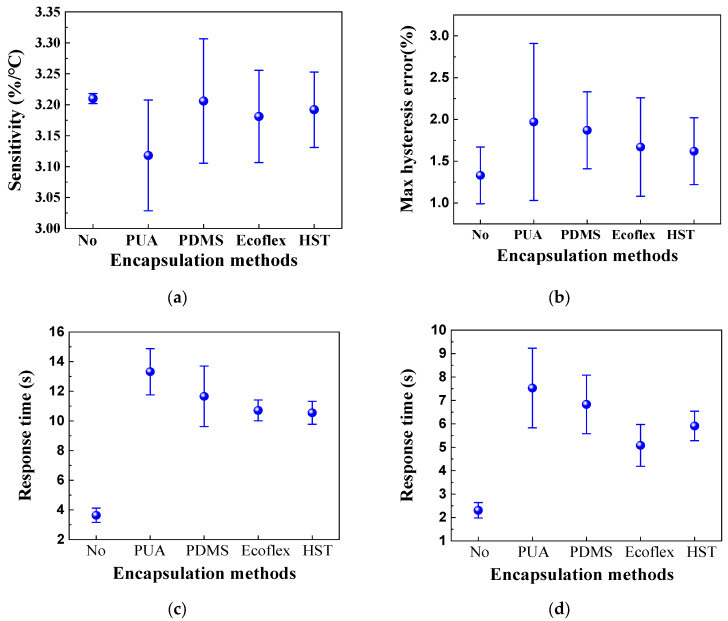
Sensing performance of HETS yarn with different encapsulation methods. (**a**) Sensitivity; (**b**) maximum hysteresis error; (**c**) natural cooling; and (**d**) heating.

The relative change in resistance, as well as the heating and natural cooling resistance and response time of HETS yarn, were measured and compared before and after encapsulation. The corresponding results are presented in Figure 12.

The results presented in Figure 12 demonstrate that the encapsulation process adversely affects the sensing performance of HETS yarn, as it hinders and delays heat transmission between the NTC thermistor and heating plate surface due to the presence of encapsulating material. This phenomenon means that HETS yarn needs a longer time to achieve the temperature balance between the thermistor and the test subject. Notably, among all the tested materials, Figure 12a shows that the yarn with PUA encapsulation exhibits the lowest sensitivity in HETS yarn, the higher max hysteresis error, and the longest response time, and then PUS is not the optimal encapsulation material. On the other hand, HETS yarn with both Ecoflex and HST encapsulations shows reduced maximum hysteresis and shorter natural cooling and heating response times compared to others. However, it is worth mentioning that the heating response time with HST is longer than that with Ecoflex. In summary, Ecoflex-encapsulated HETS yarn demonstrates a superior temperature sensing performance, and then Ecoflex is the relatively optimal encapsulation material to minimize its effect on temperature sensing performance.

## 5. Conclusions

To address the issues of poor mechanical durability and to improve the washability observed in previously reported hybrid electronic sensing yarn, this study developed a stretchable and durable hybrid electronic temperature sensing yarn by integrating a large stretchable conductive composite yarn (enameled copper wire wrapped around spandex) with an NTC thermistor. The optimal method for interconnection and encapsulation was found to be the heat-pressing soldering process using tin paste, along with Ecoflex encapsulation using a tube mold. These methods effectively insulated the bare interconnection area, improved mechanical endurance, and alleviated the stress concentration effect near the interconnection area. The sensing yarn fabricated with the heat-pressing soldering interconnection process exhibited a sensitivity of 3.210%/°C, a maximum hysteresis error of 1.33%, and a response time of less than 5 s. Furthermore, the influence of electrical resistance in enameled copper wire on sensing properties was found to be negligible. While the encapsulation structure had minimal impact on sensitivity and hysteresis, it significantly affected the response time. Future work will focus on applying the heat transfer theory to establish a deterministic relationship between the encapsulating structure and sensing performance of HETS yarn, and a systematic evaluation will be carried out on its machine washability.

## Figures and Tables

**Table 1:** Dynamic response time of three kinds of HETS yarn.

Interconnection Method	Response Time (s)	
	Cooling	Heating
Adhesive bonding	2.78 ± 0.28	2.13 ± 0.34
Tin wire	3.20 ± 0.50	1.86 ± 0.19
Tin paste	3.64 ± 0.48	2.31 ± 0.33

## Data Availability

Data is only available by asking for the corresponding author.
